# Electroantennographic Responses of *Aromia bungii* (Faldermann, 1835) (Coleoptera, Cerambycidae) to a Range of Volatile Compounds

**DOI:** 10.3390/insects10090274

**Published:** 2019-08-27

**Authors:** Giacinto S. Germinara, Marco Pistillo, Raffaele Griffo, Antonio P. Garonna, Antonella Di Palma

**Affiliations:** 1 Department of the Sciences of Agriculture, Food and Environment, University of Foggia, Via Napoli 25, 71100 Foggia (FG), Italy; 2 Plant Protection Service of Campania Region, Centro Direzionale, Isola A6, 80124 Naples, Italy; 3 Department of Agriculture, University of Naples Federico II, Via Università 100, 80055 Portici, Italy

**Keywords:** red-necked longhorn beetle, invasive species, semiochemicals, EAG, plant volatiles

## Abstract

Background: The red-necked longhorn beetle, *Aromia bungii*, is one of the most damaging pests of stone fruit trees. Native to the south-eastern Palearctic and Oriental regions, it invaded and is established to some extent in the Campania Region (Southern Italy). In several cerambycid species, volatile organic compounds (VOCs) have been shown to play a role in mate and host plant location. Methods: The electroantennographic (EAG) technique was employed to explore the antennal chemoreceptivity of male and female *A. bungii* antennae to 90 VOCs. Results: Increasing EAG amplitudes from the basal to the distal antennal segments were recorded in response to six selected plant volatiles. From the distal flagellomeres, the largest EAG responses (>0.8 mV) were elicited by 2-hexanol, octanal, sulcatone, guaiacol, sulcatol, 2,4-dimethyl-3-hexanol, 2,4-dimethyl-2-hexanone, heptanal, nonanal, (Z)-3-hexenol, and 1-heptanol in both sexes, and by linalool, (*E*)-2-heptenal, 1-octen-3-ol, (*E*)-2-octenal, 3-octanol, (*E*)-2-octen-1-ol, α-phellandrene, and α-terpinene in males. The olfactory system of both sexes proved to be sensitive to changes in stimulus concentration and compound structure. Conclusions: this study demonstrates the capability of *A. bungii* males and females to detect and discriminate among a wide range of VOCs and provides a basis for further olfactometer and field trapping experiments aimed at identifying behaviorally-active compounds useful for the implementation of semiochemical-based control strategies for this pest.

## 1. Introduction

The red-necked longhorn beetle, *Aromia bungii* (Faldermann, 1835) (Coleoptera, Cerambycidae, subfamily Cerambycinae, tribe Callichromatini), is an oligophagous wood-borer beetle of *Prunus* spp., including many cultivated stone fruit trees such as peach, apricot, plum, and cherry [1]. Additional host plants belonging to different families have been occasionally reported in China, but these observations require confirmation [1,2,3].

Native to the south-eastern Palearctic and Oriental regions, *A. bungii* was intercepted in 2008 in United Kingdom and USA among wooden pallets and in a manufacturing plant, respectively [1]. However, the first reports of this pest from host trees outside its native range date back to 2011 in Germany [4] and 2012 in Italy [5,6]. In 2013, *A. bungii* was detected in Japan where it caused substantial damage to peach orchards and Japan’s iconic cherry blossom trees [7]. In Europe, *A. bungii* was added to the European and Mediterranean Plant Protection Organization (EPPO) A1 list of quarantine pests since 2014 [8,9], and currently, it is established to some extent in the Campania Region (Southern Italy) where quarantine measures are being applied and eradication is ongoing [10,11].

*A. bungii* has one generation every 2–4 years and overwinters as larvae of different instars in tunnels excavated in the trunk and main branches of healthy trees [6,12]. Larval attacks result in a weakening of trees and decrease of fruit yield. However, serious infestation can cause tree decay also as a consequence of an increased susceptibility to diseases. In Italy, several hundred apricot, cherry, and plum trees have been severely damaged or killed by larval attack [6,13].

The endophytic development of the larvae makes early detection of the infestation difficult, but becomes evident in its late stages when larval frass accumulate at the base of the plants. Moreover, the cryptic nature of larval development makes any control measures against this stage poorly effective. Therefore, setting up an effective adult monitoring tool has a great practical interest both for *A. bungii* detection in new areas, where the population level is generally low, and to carry out timely insecticide applications against adults, eggs, and larvae at hatching.

Studies on the *A. bungii* intraspecific communication have identified (*E*)-2-cis-6,7-epoxynonenal as the main male-produced sex-aggregation pheromone that was attractive to both sexes in field experiments [7]. In further field trapping tests, trap catches were significantly increased by adding (*E*,*Z*)-2,6-nonadienal to the main component of the male sex-aggregation pheromone [14]. Moreover, (*R*)-(+)-citronellal, previously detected from the body of *A. bungii* females [15], was shown to elicit male attraction in Y-tube olfactometer bioassays and it has been proposed as a female-produced sex pheromone [16].

Phytophagous insects use plant volatiles to locate suitable feeding, mate, and oviposition sites [17,18]. In several cerambycid species, plant volatiles, attractive to both sexes, bring males and females together on a larval host, thus suggesting a role in mate location. Moreover, adults of some cerambycid species are capable of discriminating between odors of stressed and healthy trees or between volatiles of host and non-host plants, also indicating a role of plant volatiles in host location [19,20,21,22,23,24,25,26]. In some species, synergism between plant volatiles and pheromones has been demonstrated [26,27,28,29,30,31,32]. Moreover, plant volatiles have been exploited in monitoring traps, alone or in combination with insect pheromones [19,21,29,33,34]. 

Insects perceive many odors with receptors located on the antennae and measurements of the electrical signals associated with olfaction, using the electroantennographic technique (EAG), have been widely used in the search for putative behaviorally-active compounds. In fact, an EAG response, the summation of receptor potentials evoked by a test stimulus from the antenna [35], represents the antennal olfactory sensitivity to the compound tested, and EAG-active compounds are frequently of ecological significance [36].

In *A. bungii*, the EAG technique has been used in combination with gas chromatography (GC) to identify volatile compounds present in male and female extracts that are able to elicit an antennal response in conspecific adults [7] and to evaluate the antennal responsiveness of males to (*R*)-(+)-citronellal [16]. However, no studies have been carried out to evaluate the olfactory sensitivity of *A. bungii* adults to plant volatiles.

At least three types of sensilla basiconica on adult *A. bungii* antennae with a putative olfactory function have been described [37]. In both sexes, these sensilla are present in all flagellomeres but they are mainly concentrated in two lateral bands facing the abaxial surface of the antennae. These bands are thinner in the first to fifth flagellomeres while they increase in size in the more distal flagellomeres, especially the last one (ninth).

In the present study, the EAG technique was employed to explore the antennal chemoreceptivity of male and female *A. bungii* antennae to 90 volatile organic compounds (VOCs). The EAG responses of male and female specimens were also compared. The elucidation of the *A. bungii* sensitivity profile to a variety of VOCs provides a basis for future investigations aimed at identifying behaviorally-active compounds.

## 2. Materials and Methods 

### 2.1. Insects

Logs with *A. bungii* larvae were collected from infested apricot trees in the Marigliano (Naples, Campania, Italy) area and held in rearing cages until adult emergence. Emerged adults were collected daily and placed individually in transparent plastic containers (6 cm diameter × 8 cm height) covered with screw caps with a central hole (2 cm) screened by a metallic net (mesh size 1 mm) to allow air exchange. Insects were maintained at 25 ± 2 °C and 60 ± 5% relative humidity in a 16:8 light: day photoperiod and fed with apple pieces that were renewed every 3 days. For the EAG tests, 10–15-day-old unmated males and females were used. Before EAG experiments, insects were kept in the absence of food odors for at least 4 h.

### 2.2. Odor Stimuli

Test compounds were 90 VOCs selected to represent different chemical categories including aliphatic alcohols, aldehydes, esters, and ketones, terpenoids, and aromatics (Table 1) (Sigma-Aldrich, Milan, Italy). For each test compound, a 100 μg/μL mineral oil (Sigma-Aldrich, Milan, Italy) solution was prepared. To calculate dose-response curves, mineral oil decimal solutions (0.001–100 μg/μL) of the green leaf volatile (*Z*)-3-hexenol, used as a standard, were also prepared. Solutions were stored at −20 °C until needed. Before each EAG experiment, 10 μL of each test solution were applied to filter paper (Whatman No. 1) strips (1 cm^2^) inserted in a Pasteur pipette (15 cm long) and used as an odor cartridge.

### 2.3. Electroantennography (EAG)

The chemoreceptivity of male and female *A. bungii* antennae to the selected VOCs was examined using the EAG technique. In the first set of experiments, the EAG responses of the basal (first to third), middle (fourth to sixth), and distal (seventh to ninth) groups of flagellomeres to 1 mg loads (10 µL of 100 µg/µL mineral oil solution) of six VOCs ((*Z*)-3-hexenol, limonene, sulcatol, sulcatone, nonanal, 2-hexanone) were evaluated to select the most sensitive portion of the antenna. Antennal portions (basal, middle, distal) were amputated at the base of their first flagellomere and at the end of their terminal flagellomere, except for the distal portion whose terminal flagellomere (ninth) was left uncut. In a second set of experiments, the EAG responses of flagellomeres seven to nine to different concentrations (0.001, 0.01, 0.1, 1, 10, 100 μg/μL) of (*Z*)-3-hexenol were measured to evaluate olfactory sensitivity. In a third series of experiments, the EAG responses of flagellomeres seven to nine to 1 mg loads of test VOCs were determined to evaluate adult olfactory selectivity. 

The base of each antennal portion was inserted into a glass pipette containing Kaissling saline solution [38] used as the neutral electrode, while the distal end was put in contact with the end of a similar pipette that served as the recording electrode. The electrical continuity between the antennal preparation and an AC/DC UN-6 amplifier in DC mode connected to a PC equipped with the EAG 2.0 program (Syntech Laboratories, Hilversum, The Netherlands) was maintained using AgCl-coated silver wires. A flow of charcoal-filtered humidified air (500 mL/min) was directed constantly onto the antennal portions through a stainless-steel delivery tube (1 cm i.d.) with the outlet at nearly 1 cm from the antenna. 

Based on previous scanning electron microscopy (SEM) observations [37], reporting the presence of sensilla basiconica with a putative olfactory function mainly on the abaxial surface, each antennal portion was exposed to the air flow accordingly. Over 1 s, 2.5 cm^3^ of vapor from an odor cartridge were blown into the air stream using a disposable syringe. Stimuli were randomly sequenced in the first and second set of experiments whereas they were applied in ascending concentrations in dose-response experiments. Ten µL of mineral oil (control stimulus) and 10 μL of a 10 μg/μL (*Z*)-3-hexenol mineral oil solution (standard stimulus) were presented at the beginning of the experiment and after each group of five test compounds. Intervals between stimuli were 1 min. Each compound was tested on five antennae of different insects of each sex.

### 2.4. Data Analysis

The maximum amplitude of negative polarity deflection (−mV) induced by a stimulus was used to measure the EAG response [39]. The absolute EAG response (mV) to each test stimulus was subtracted by the mean response to the two nearest mineral oil controls in order to compensate for solvent and mechanosensory artifacts [40]. The resultant EAG value was corrected based on the reduction of the EAG amplitude to the standard stimulus to compensate for the decline of the antennal responsiveness during the experiment [41]. 

The corrected EAG responses to each compound were compared to a “0” value using the Wilcoxon rank sum test and considered measurable if significant at *p* = 0.05. The mean EAG responses of the three antennal portions of each sex to the six compounds tested were subjected to analysis of variance (ANOVA) followed by the Tukey’s HSD (Honestly Significant Difference) test (*p* = 0.05) for mean comparison. Prior to these analyses, values were √ ×-transformed and tested for homogeneity of variance using Levene’s test. The mean male and female EAG responses to the test compound were compared using the Student’s *t*-test (*p* = 0.05) for independent samples. In dose-response curves, the activation threshold was considered to be the first dose at which the mean response was higher than a “0” value using the Shapiro-Wilk test for normality followed by the one-sample Student’s *t*-test (*p* = 0.05) [42]; the saturation level was taken as the lowest dose at which the mean response was equal to or less than the previous dose [43]. Statistical analyses were done using SPSS (Statistical Package for the Social Sciences) version 10.0.7 for Windows (SPSS Inc., Chicago, IL, USA).

## 3. Results

### 3.1. Chemoreceptivity of Flagellomeres

**Figure 1:**
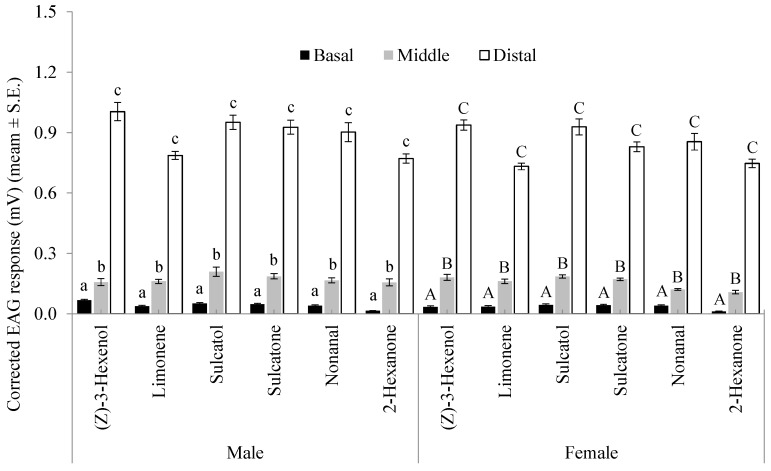
EAG responses of different groups of male and female *A. bungii* flagellomeres (basal, first to third; middle, fourth to sixth; distal, seventh to ninth) to 1 mg loads of six VOCs.

On stimulation with the 1 mg dose of (*Z*)-3-hexenol, limonene, sulcatol, sulcatone, nonanal, and 2-hexanone, measurable EAG responses were elicited in the basal (first to third), middle (fourth to sixth), and distal (seventh to ninth) flagellomeres of *A. bungii* males and females (*p* < 0.05 in all Wilcoxon rank sum tests). For all these six compounds, there were significant differences among the EAG responses of the different flagellum portions both in males (F = 432.45–1118.76, df = 2; *p* < 0.001) and females (F = 628.77–1250.14, df = 2, *p* < 0.001). The mean EAG responses of the distal flagellomeres were significantly higher than those of the middle flagellomeres, which were significantly more responsive than the basal ones (Figure 1).

### 3.2. Sensitivity of Distal Flagellomeres

**Figure 2:**
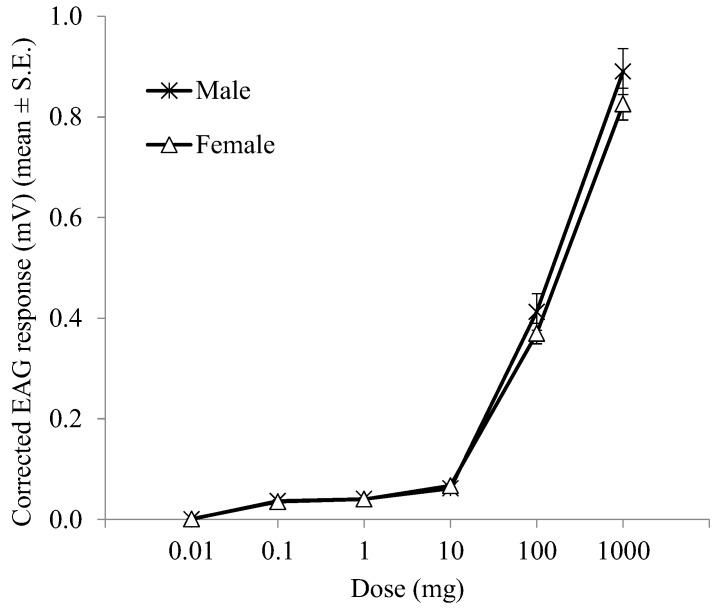
EAG dose-response curves of male and female *A. bungii* distal flagellomeres (seventh to ninth) to ascending doses of (*Z*)-3-hexenol.

The sensitivity of the distal male and female *A. bungii* flagellomeres to ascending doses of (*Z*)-3-hexenol is reported in Figure 2. In the range of doses tested, the mean EAG amplitude varied from 0.001 ± 0.001 mV to 0.826 ± 0.045 mV in females and from 0.0004 ± 0.001 mV to 0.891 ± 0.032 mV in males. In both sexes, the activation threshold was 0.1 μg (*p* < 0.05; one-sample *t*-test). Male and female EAG responses increased from the 100 to 1000 μg doses indicating that no saturation of olfactory receptors occurred at the 100 µg dose. For all doses tested, there were no significant differences (*t* = −1.150 to 1.171, df = 8, *p* > 0.05; independent sample Student’s *t*-test) between the mean EAG responses of the terminal male and female flagellomeres.

### 3.3. Selectivity of Distal Flagellomeres

The mean EAG responses evoked from the distal (seventh to ninth) flagellomeres of *A. bungii* males and females by different stimuli tested are shown in Table 1. In both sexes, all compounds elicited measurable EAG responses (*p* < 0.05 in all Wilcoxon rank sum tests) ranging from 0.028 ± 0.003 mV ((*E*,*E*)-2,4-hexadienal) to 1.197 ± 0.067 mV ((*E*)-2-hexenol) in males and from 0.019 ± 0.001 mV (ethyl acetate) to 0.979 ± 0.057 mV ((*E*)-2-hexenol) in females.

The largest EAG responses (> 0.8 mV) were elicited by 2-hexanol, octanal, sulcatone, guaiacol, sulcatol, 2,4-dimethyl-3-hexanol, 2,4-dimethyl-2-hexanone, heptanal, nonanal, (*Z*)-3-hexenol, and 1-heptanol in both sexes, and by linalool, (*E*)-2-heptenal, 1-octen-3-ol, (*E*)-2-octenal, 3-octanol, (*E*)-2-octen-1-ol, α-phellandrene, and α-terpinene in males.

The weakest antennal stimulants (< 0.10 mV) were (*E,E*)-2,4-hexadienal, 1-ethanol, 2-methyl-1-butanol, 2,3-butandiol, glycerol, 2,3-butanedione, ethyl acetate, and phytol in both sexes; α-farnesene in females; and geranyl linalool, α-humulene, maltol, furfural, and vanillin in males. The mean male EAG amplitude was significantly higher than that of females for (*E*)-2-heptenal, 3-pentanol, 2-pentanone, 2-nonanone, 2-decanone, 2-undecanone, ethyl acetate, neryl acetate, phytol, eugenol, phenethyl alcohol, and γ-nonalactone (*t* = 2.33–5.54, df = 8, *p* = 0.048–0.007). In contrast, female responses were significantly higher than those of males for hexanal, (*E,E*)-2,4-hexadienal, 1-hexenol, β-pinene, and maltol (*t* = 2.57–9.00, df = 8, *p* = 0.032–0.001) (Table 1).

**Figure 3:**
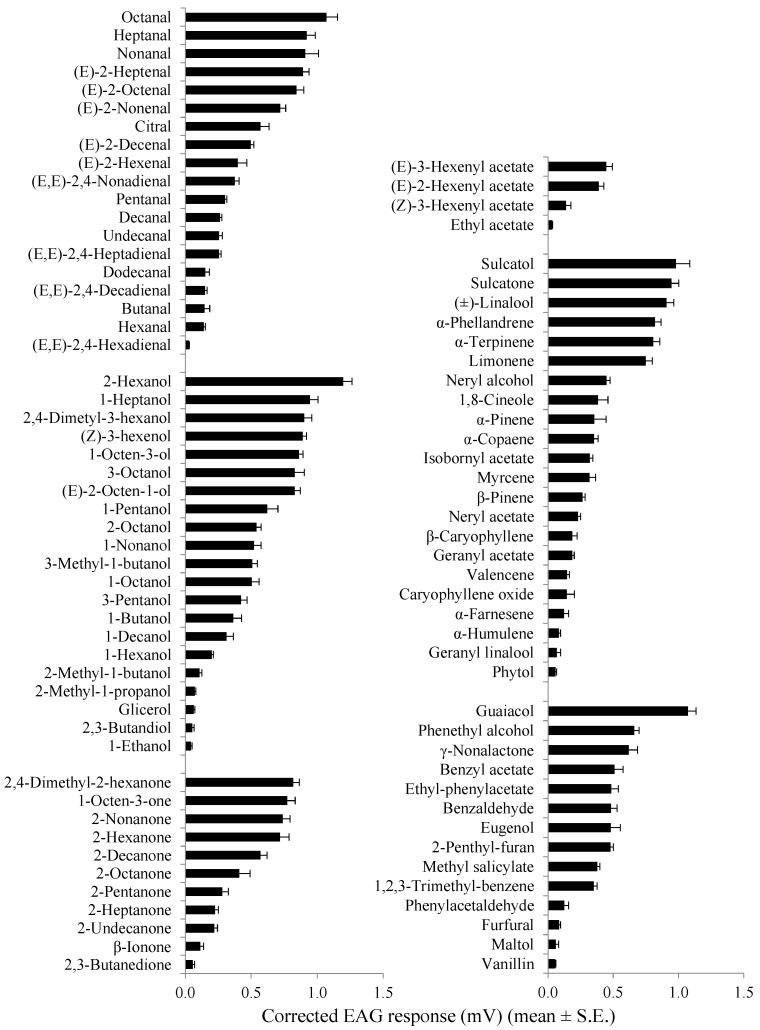
EAG response profile of male *A. bungii* distal flagellomeres (seventh to ninth) to a range of VOCs. Within each chemical group, compounds are listed according to the mean amplitude of the EAG response (n = 5) elicited at the 1 mg dose.

**Figure 4:**
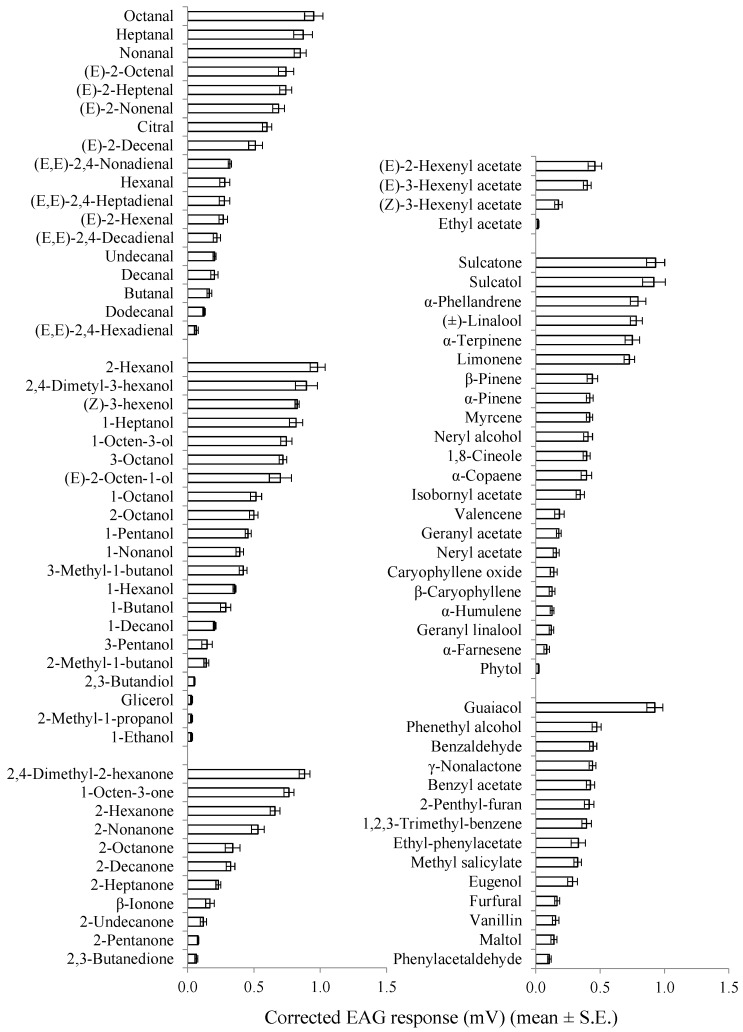
EAG response profile of female *A. bungii* distal flagellomeres (seventh to ninth) to a range of VOCs. Within each chemical group, compounds are listed according to the mean amplitude of the EAG response (n = 5) elicited at the 1 mg dose.

In both sexes, octanal, 2-hexanol, 2,4-dimethyl-2-hexanone, (*E*)-3-hexenhyl acetate, sulcatol, and guaiacol were the strongest antennal stimulants within the chemical groups of aliphatic aldehydes, alcohols, ketones, acetates, terpenoids, and aromatics, respectively (Figure 3 and Figure 4).

Within saturated aliphatic aldehydes, alcohols, and ketones, compounds with shorter carbon chain lengths (C_2_–C_5_) elicited lower EAG amplitudes in males than compounds with higher carbon chain lengths (C_6_–C_9_). Similarly, butanal, 1-ethanol, and 2-pentanone evoked female EAG responses that were lower than those induced by compounds with longer carbon chains in the corresponding chemical classes. Considering the antennal responsiveness to C_6_, C_7_, and C_9_ aliphatic aldehydes, diunsaturated compounds elicited lower EAG responses than monounsaturated analogs, and the latter were weaker antennal stimulants than the corresponding saturated aldehydes (Figure 3 and Figure 4).

## 4. Discussion

Previous SEM observations [37] showed that both male and female *A. bungii* antennae contain different types of sensilla basiconica with a putative olfactory function, mainly concentrated in two lateral bands facing the abaxial surface and that these bands are growing in width from the base to the apex of the antenna. In the present study, EAG preparations set up by exposing the abaxial surface of three antennal portions to the air stream from an EAG apparatus enabled us to get measurable EAG recordings on stimulation with different VOCs. The amplitudes of EAG responses elicited by six selected compounds in the distal, middle, and basal portions of the antennae were respectively high, intermediate, and low in both sexes, consistent with the increasing width of the bands containing sensilla basiconica and thus supporting the involvement of sensilla basiconica in olfaction. Increasing EAG responses to plant volatiles from the basal to the distal antennal segments have also been reported for the cerambycid *Batocera horsfieldi* (Hope) [44] and probably results from both natural and sexual selection that have shaped antennal morphology to optimize odor perception [45].

The antennal chemoreceptivity toward different volatile compounds has been investigated in a number of cerambycid species [46,47,48,49]; however, to our knowledge, this is the first study exploring the antennal sensitivity of a member of the Callichromatini tribe to a wide variety of VOCs.

In both sexes, the amplitude of EAG responses did not correlate well with the volatility of compounds, as shown by the EAG profiles of compounds with increasing carbon chain length and different unsaturation, within the functional-group classes of aliphatic aldehydes, alcohols and ketones. In these three groups, in fact, highly volatile compounds with shorter carbon chain lengths elicited lower EAG responses than less volatile compounds with longer carbon chain lengths. In the same way, diunsaturated aldehydes were weaker antennal simulants than saturated analogs. This clearly shows the importance of the sensitivity of the peripheral olfactory system of *A. bungii* adults rather than the volatility of compounds in determining differences in the amplitude of EAG responses to different VOCs. These results, which imply the ability of adult *A. bungii* to discriminate among different chemical cues, are fairly consistent with those reported in many previous EAG studies with different insect species [18,36,42] and support the paradigm that highly EAG-active compounds may hold some ecological significance. As shown by the EAG responses recorded on stimulation with increasing concentrations of the ubiquitous green leaf volatile (*Z*)-3-hexen-1-ol, antennae of both sexes proved to be sensitive to stimulus variations in a wide range of doses and support the possibility that plant odors may act as long-distance cues to *A. bungii* adults.

A large degree of similarity between the EAG responses of males and females was observed with only 17 of the 90 compounds tested eliciting significant differences. Male responses exceeded those of females for 12 compounds, whereas for 5 compounds, responses of females were significantly higher than those of males. Among cerambycids, sexually dimorphic EAG responses have also been reported in *B. horsfieldi* [49]; this could be due to differences in the number of antennal olfactory neurons tuned to individual compounds and/or to qualitative physiological differences in olfaction [50] and more likely reflects differences in the role played by the same VOC in the ecology of males and females.

Despite these differences, the EAG profiles of male and female responses to compounds tested were similar. In this regard, it is worth noting that 2-hexanol, octanal, guaiacol, sulcatol, and sulcatone were, in decreasing order, the strongest antennal stimulants in both sexes. All these compounds have been reported as plant volatiles and/or insect secretion components and have different infochemical functions in insects [51]; however, as yet, very little is known about their behavioral function in cerambycids. For instance, 2-hexanol has been identified as a component of a plant volatile blend attractive to adults of the plum curculio, *Conotrachelus nenuphar* (Herbst) [52], and it is also a component of the alarm pheromone of male meliponine bees that induce fighting in workers and flight in males [53]. In Coleoptera, octanal was identified in blends with kairomonal effects to *Aethina tumida* (Murray) [54], *Oryzaephilus surinamensis* (L.) [55], and *Listronotus maculicollis* (Kirby) [56], but it is also part of the defense secretions of some staphylinid species [57].

Among the bark volatiles of some angiosperms, guaiacol has been found to be EAG-active for five species of coniferous bark beetles and it might act as a non-host cue in the host selection process by these species [58]. In addition, it is an aggregation pheromone component in both the adult and nymph desert locust, *Schistocerca gregaria* (Forskål) [59], and in the blend of larval frass volatiles repelling oviposition in *Cydalima perspectalis* (Walker) [60]. Sulcatol is the aggregation pheromone of several scolytid beetles [61,62,63]. Sulcatol and sulcatone in a naturally occurring mixture with 2-tridecanone mediate spacing behavior of *Rhopalosiphum padi* (L.) feeding on cereals [64]. Moreover, these two compounds are released by males of the ambrosia beetles *Megaplatypus mutatus* (Chapuis) and *Platypus cylindrus* (F.) (Coleoptera, Platypodidae) and attract females and males [65,66]. 

## 5. Conclusions

In conclusion, this study provides electrophysiological support for the putative chemoreceptivity of sensilla basiconica previously described in *A. bungii* adults and demonstrates the capability of antennae of both sexes to detect and discriminate among a wide range of VOCs. Obviously, the EAG response of an antenna to a particular compound provides no insight into behavioral response and EAG-active compounds may mediate behavioral responses other than attraction and may even be repellent. Considering the oligophagy of *A. bungii* and its preference for healthy host plants, it seems possible that detection of a large number of compounds by adults would allow for both an effective selection of host plants, in different physiological states, and non-host avoidance, as also suggested for some bark beetles [58,67] and insects with a limited range of host plants [68,69,70]. From this perspective, this study provides a basis for future olfactometer bioassays and field trapping experiments aimed at identifying behaviorally-active compounds useful to develop semiochemical-based control strategies for this pest.

## Figures and Tables

**Table 1:** EAG responses of male and female *A. bungii* distal flagellomeres (seventh to ninth) to 90 volatile organic compounds (VOCs).

*Class* Compound ^a^	Chemical Purity (%)	Corrected EAG Response in mV (Mean ± SE)	
		Males	Females
*Aliphatic aldehydes*			
Butanal	99.0	0.15 ± 0.04	0.16 ± 0.02
Pentanal	95.0	0.30 ± 0.01	0.27 ± 0.02
Hexanal **	98.0	0.14 ± 0.01	0.28 ± 0.04
Heptanal	95.0	0.92 ± 0.07	0.87 ± 0.07
Octanal	99.0	1.07 ± 0.08	0.95 ± 0.07
Nonanal	95.0	0.91 ± 0.10	0.85 ± 0.04
Decanal	95.0	0.26 ± 0.01	0.20 ± 0.03
Undecanal	97.0	0.26 ± 0.03	0.20 ± 0.01
Dodecanal	92.0	0.15 ± 0.03	0.12 ± 0.01
(*E*)-2-Hexenal	99.0	0.40 ± 0.07	0.27 ± 0.03
(*E*)-2-Heptenal *	97.0	0.89 ± 0.05	0.74 ± 0.04
(*E*)-2-Octenal	94.0	0.84 ± 0.05	0.74 ± 0.06
(*E*)-2-Nonenal	97.0	0.72 ± 0.04	0.69 ± 0.04
(*E*)-2-Decenal	95.0	0.50 ± 0.02	0.51 ± 0.05
(*E,E*)-2,4-Hexadienal *	95.0	0.03 ± 0.01	0.07 ± 0.01
(*E,E*)-2,4-Heptadienal	88.0	0.26 ± 0.02	0.28 ± 0.04
(*E,E*)-2,4-Nonadienal	85.0	0.38 ± 0.03	0.32 ± 0.01
(*E,E*)-2,4-Decadienal	85.0	0.15 ± 0.02	0.22 ± 0.03
Citral	95.0	0.57 ± 0.07	0.60 ± 0.03
*Aliphatic alcohols*			
1-Ethanol	99.8	0.04 ± 0.01	0.03 ± 0.01
1-Butanol	99.0	0.36 ± 0.06	0.29 ± 0.04
1-Pentanol	99.0	0.62 ± 0.08	0.46 ± 0.02
3-Pentanol **	98.0	0.42 ± 0.05	0.15 ± 0.04
1-Hexanol **	98.0	0.20 ± 0.01	0.35 ± 0.01
2-Hexanol	99.0	1.20 ± 0.07	0.98 ± 0.06
1-Heptanol	98.0	0.94 ± 0.06	0.82 ± 0.05
1-Octanol	98.0	0.50 ± 0.06	0.52 ± 0.04
2-Octanol	96.0	0.54 ± 0.04	0.50 ± 0.03
3-Octanol	99.0	0.83 ± 0.07	0.72 ± 0.03
1-Octen-3-ol	98.0	0.86 ± 0.03	0.74 ± 0.04
1-Nonanol	98.0	0.52 ± 0.05	0.39 ± 0.03
1-Decanol	98.0	0.31 ± 0.05	0.20 ± 0.01
(*Z*)-3-Hexenol	98.0	0.89 ± 0.03	0.83 ± 0.05
(*E*)-2-Octen-1-ol	97.0	0.83 ± 0.04	0.70 ± 0.08
2-Methyl-1-butanol	99.0	0.07 ± 0.01	0.03 ± 0.01
2-Methyl-1-propanol	99.5	0.11 ± 0.02	0.14 ± 0.02
3-Methyl-1-butanol	99.0	0.51 ± 0.04	0.42 ± 0.03
2,4-Dimetyl-3-hexanol	99.0	0.90 ± 0.06	0.90 ± 0.90
2,3-Butandiol	98.0	0.05 ± 0.01	0.05 ± 0.01
Glicerol	99.0	0.06 ± 0.01	0.03 ± 0.01
*Aliphatic ketones*			
2-Pentanone **	97.0	0.28 ± 0.05	0.08 ± 0.01
2-Hexanone	99.0	0.72 ± 0.07	0.66 ± 0.04
2-Heptanone	98.0	0.22 ± 0.03	0.23 ± 0.02
2-Octanone	98.0	0.41 ± 0.08	0.34 ± 0.06
2-Nonanone *	99.0	0.74 ± 0.05	0.53 ± 0.05
2-Decanone **	98.0	0.57 ± 0.05	0.32 ± 0.03
2-Undecanone *	99.0	0.22 ± 0.03	0.12 ± 0.02
1-Octen-3-one	96.0	0.77 ± 0.06	0.76 ± 0.04
β-Ionone	95.0	0.11 ± 0.02	0.17 ± 0.03
2,4-dimetyl-2-hexanone	98.0	0.82 ± 0.04	0.88 ± 0.04
2,3-Butanedione	97.0	0.06 ± 0.01	0.06 ± 0.01
*Aliphatic esters*			
Ethyl acetate *	99.0	0.03 ± 0.01	0.02 ± 0.01
(*E*)-2-Hexenyl acetate	98.0	0.39 ± 0.04	0.46 ± 0.05
(*E*)-3-Hexenyl acetate	98.0	0.45 ± 0.05	0.40 ± 0.03
(*Z*)-3-Hexenyl acetate	98.0	0.14 ± 0.04	0.18 ± 0.03
*Terpenes*			
α-Pinene	98.0	0.35 ± 0.09	0.42 ± 0.03
β-Pinene **	98.0	0.26 ± 0.02	0.44 ± 0.04
Limonene	97.0	0.75 ± 0.05	0.73 ± 0.04
α-Farnesene	95.0	0.12 ± 0.04	0.08 ± 0.02
β-Caryophyllene	80.0	0.19 ± 0.04	0.13 ± 0.02
Myrcene	92.0	0.32 ± 0.05	0.42 ± 0.02
α-Terpinene	95.0	0.81 ± 0.05	0.75 ± 0.06
1.8-Cineole	99.0	0.38 ± 0.08	0.40 ± 0.03
(±)-Linalool	99.0	0.91 ± 0.06	0.78 ± 0.05
Sulcatone (6-Methyl-5-hepten-2-one)	99.0	0.95 ± 0.06	0.91 ± 0.07
Sulcatol (6-Methyl-5-hepten-2-ol)	99.0	0.98 ± 0.11	0.92 ± 0.09
Neryl alcohol	98.0	0.45 ± 0.03	0.41 ± 0.03
Neryl acetate *	98.0	0.23 ± 0.02	0.16 ± 0.02
Geranyl acetate	97.0	0.18 ± 0.02	0.18 ± 0.02
Isobornyl acetate	95.0	0.32 ± 0.02	0.35 ± 0.03
α-Phellandrene	95.0	0.82 ± 0.05	0.79 ± 0.06
Geranyl linalool	95.0	0.07 ± 0.03	0.12 ± 0.02
Phytol *	97.0	0.06 ± 0.01	0.02 ± 0.01
α-Copaene	90.0	0.35 ± 0.03	0.39 ± 0.04
Valencene	70.0	0.15 ± 0.02	0.18 ± 0.04
α-Farnesene	95.0	0.12 ± 0.04	0.08 ± 0.02
β-Caryophyllene	80.0	0.19 ± 0.04	0.13 ± 0.02
Caryophyllene oxide	95.0	0.14 ± 0.06	0.14 ± 0.03
α-Humulene	96.0	0.08 ± 0.01	0.13 ± 0.01
*Aromatics*			
Guaiacol	98.0	1.07 ± 0.06	0.93 ± 0.06
Eugenol *	99.0	0.48 ± 0.07	0.29 ± 0.04
Maltol *	99.0	0.06 ± 0.02	0.14 ± 0.02
Phenethyl alcohol **	99.0	0.66 ± 0.04	0.47 ± 0.04
Furfural	99.0	0.08 ± 0.01	0.17 ± 0.02
Phenylacetaldehyde	90.0	0.12 ± 0.03	0.11 ± 0.01
Vanillin	99.0	0.06 ± 0.01	0.15 ± 0.03
Benzaldehyde	99.0	0.48 ± 0.05	0.45 ± 0.03
Methyl salicylate	99.0	0.38 ± 0.02	0.33 ± 0.03
1,2,3-Trimethyl-benzene	90.0	0.35 ± 0.03	0.39 ± 0.04
γ-Nonalactone *	98.0	0.62 ± 0.07	0.44 ± 0.03
2-Penthyl-furan	98.0	0.48 ± 0.02	0.41 ± 0.04
Benzyl acetate	99.0	0.51 ± 0.07	0.42 ± 0.03
Ethyl-phenylacetate	98.0	0.48 ± 0.05	0.33 ± 0.06

^a^ Asterisks indicate significant differences between sexes (* *p* = 0.05, ** *p* = 0.01, *t*-test).

## References

[1] CABI (2019). *Aromia* *bungii*. Invasive Species Compendium.

[2] Hua L.Z. (2002). List of Chinese Insects.

[3] Smith J.W. (2009). NPAG report. Aromia bungii (Faldermann): Redneck Longhorned Beetle Coleoptera/Cerambycidae.

[4] EPPO (2012). First Report of *Aromia bungii* in Germany: Addition to the EPPO Alert List. EPPO Reporting Service.

[5] EPPO (2012). First Report of *Aromia bungii* in Italy. EPPO Reporting Service.

[6] Garonna A.P., Nugnes F., Epinosa B., Griffo R., Benchi D. (2013). *Aromia bungii*, nuovo tarlo asiatico ritrovato in Campania (*Aromia bungii*, a new Asian worm found in Campania). Inf. Agrar..

[7] Xu T., Yasui H., Teale S.A., Fujiwara-Tsujii N., Wickham J.D., Fukaya M., Hansen L., Kiriyama S., Hao D., Nakano A. (2017). Identification of a male-produced sex-aggregation pheromone for a highly invasive cerambycid beetle, *Aromia bungii*. Sci. Rep..

[8] EPPO (2014). Pest Risk Analysis for Aromia bungii.

[9] Cocquempot C. (2015). *Aromia bungii*. EPPO datasheet on pests recommended for regulation. EPPO Bull..

[10] Nugnes F., Russo E., Garonna A.P., Griffo R. L’attività di monitoraggio ed eradicazione di *Aromia bungii* (Faldermann) in Campania. Proceedings of the XXIV Congresso Nazionale Italiano di Entomologia.

[11] European Union (2018). Commission Implementing Decision (EU) 2018/1503 of 8 October 2018 as regards measures to prevent the introduction into and the spread within the Union of *Aromia bungii* (Faldermann). Off. J. EU.

[12] Gressitt J.L. (1942). Destructive Long-Horned Beetle borers at Canton, China. Special Publication 1. Lingnan Natural History Survey and Museum.

[13] Carella D. (2019). Piano d’azione regionale per la lotta al Cerambicide Aromia bungii-VI aggiornamento; Decreto Dirigenziale n. 31. *Bollettino Ufficiale della Regione Campania*, Italy.

[14] Zou Y., Hansen L., Xu T., Teale S.A., Hao D., Millar J.G. (2019). Optimizing pheromone-based lures for the invasive red-necked longhorn beetle, *Aromia bungii*. J. Pest Sci..

[15] Wei J.R., Liu X.B., Niu Y.L., Wang J.J. (2013). Identification of volatiles released from the living adult *Aromia bungii* Faldermann. For. Pest Dis..

[16] Wang W.C., Cao D.D., Jin M., Wei J.R. (2018). (*R*)-(+)-citronellal identified as a female-produced sex pheromone of *Aromia bungii* Faldermann (Coleoptera: Cerambycidae). Egypt. J. Biol. Pest Control.

[17] Dickens J.C. (1984). Olfaction in the boll weevil *Anthonomus grandis* Boh (Coleoptera: Curculionidae): Electroantennogram studies. J. Chem. Ecol..

[18] Visser J.H., Piron P.G.M., Hardie J. (1996). The aphids’ peripheral perception of plant volatiles. Entomol. Exp. Appl..

[19] Hanks L.M. (1999). Influence of the larval host plant on reproductive strategies of cerambycid beetles. Annu. Rev. Entomol..

[20] Reagel P.F., Ginzel M.D., Hanks L.M. (2002). Aggregation and mate location in the red milkweed beetle (Coleoptera: Cerambycidae). J. Insect Behav..

[21] Allison J.D., Borden J.H., Seybold S.J. (2004). A review of the chemical ecology of the Cerambycidae (Coleoptera). Chemoecology.

[22] Wang Q., Chen L.Y. (2005). Mating behavior of a flower visiting longhorn beetle *Zorion guttigerum* (Westwood) (Coleoptera: Cerambycidae: Cerambycinae). Naturwissenschaften.

[23] Li J.G., Jin Y.J., Luo Y.Q., Xu Z.C., Chen H.J. (2003). Leaf volatiles for host tree *Acer negundo*: Diurnal rhythm and behavior responses of *Anoplophora glabripennis* to volatiles in field. Acta Bot. Sin..

[24] Nehme M.E., Keena M.A., Zhang A., Baker T.C., Hoover K. (2009). Attraction of *Anoplophora glabripennis* to male-produced pheromone and plant volatiles. Environ. Entomol..

[25] Collignon R.M., Swift I.P., Zou Y., McElfresh J.S., Hanks L.M., Millar J.G. (2016). The influence of host plant volatiles on the attraction of longhorn beetles to pheromones. J. Chem. Ecol..

[26] Millar J.G., Hanks L.M., Wang Q. (2017). Chemical ecology of Cerambycids. Cerambycidae of the World: Biology and Pest Management.

[27] Nakamuta K., Leal W.S., Nakashima T., Tokoro M. (1997). Increase of trap catches by a combination of male sex pheromones and floral attractant in longhorn beetle, *Anaglyptus subfasciatus*. J. Chem. Ecol..

[28] Silk P.J., Lemay M.A., LeClair G., Sweeney J., Magee D. (2010). Behavioral and electrophysiological responses of *Tetropium fuscum* (Coleoptera: Cerambycidae) to pheromone and spruce volatiles. Environ. Entomol..

[29] Sweeney J., De Groot P., Macdonald L., Smith S., Cocquempot C., Kenis M., Gutowski J.M. (2004). Host volatile attractants and traps for detection of *Tetropium fuscum* (F.), *Tetropium castaneum* (L.) and other longhorned beetles (Coleoptera: Cerambycidae). Environ. Entomol..

[30] Fujiwara-Tsujii N., Yasui H., Wakamura S., Hashimoto I., Minamishima M. (2012). The white-spotted longicorn beetle, *Anoplophora malasiaca* (Coleoptera: Cerambycidae), with blueberry as host plant, utilizes host chemicals for male orientation. Appl. Entomol. Zool..

[31] Yasui H. (2009). Chemical communication in mate location and recognition in the white-spotted longicorn beetle, *Anoplophora malasiaca* (Coleoptera: Cerambycidae). Appl. Entomol. Zool..

[32] Yasui H., Fujiwara-Tsujii N., Wakamura S. (2011). Volatile attractant phytochemicals for a population of white-spotted longicorn beetles *Anoplophora malasiaca* (Thomson) (Coleoptera: Cerambycidae) fed on willow differ from attractants for a population fed on citrus. Chemoecology.

[33] Ibeas F., Gallego D., Diez J.J., Pajares J.A. (2007). An operative kairomonal lure for managing pine sawyer beetle *Monochamus galloprovincialis* (Coleoptera: Cerambycidae). J. Appl. Ent..

[34] Miller D.R., Asaro C., Crowe C.M., Duerr D.A. (2011). Bark beetle pheromones and pine volatiles: Attractant kairomone lure blend for longhorn beetles (Cerambycidae) in pine stands of the southeastern United States. J. Econ. Entomol..

[35] Schneider D. (1962). Electrophysiological investigations on the olfactory specificity of sexual attracting substances in different species of moth. J. Insect Physiol..

[36] Van Der Pers J.N.C. (1981). Comparison of electroantennogram response spectra to plant volatiles in seven species of *Yponomeuta* and in the torticid *Adoxophyes orana*. Entomol. Exp. Appl..

[37] Di Palma A., Pistillo M., Griffo R., Garonna A.P., Germinara G.S. (2019). Scanning electron microscopy of the antennal sensilla and their secretion analysis in adults of *Aromia bungii* (Faldermann, 1835) (Coleoptera, Cerambycidae). Insects.

[38] Kaissling K.E., Thorson J., Sattelle D.B., Hall L.M., Hildebrand J.G. (1980). Insect olfactory sensilla: Structural, chemical and electrical aspects of the functional organization. Receptors for Neurotransmitters, Hormones and Pheromones in Insects.

[39] Light M.D., Kamm J.A., Buttery R.G. (1992). Electroantennogram response of alfalfa seed chalcid, *Bruchophagus roddi* (Hymenoptera: Eurytomidae) to host- and non host-plant volatiles. J. Chem. Ecol..

[40] Raguso R.A., Light D.M. (1998). Electroantennogram responses of male *Sphinx perelegans* hawkmoths to floral and ‘green leaf volatiles’. Entomol. Exp. Appl..

[41] Den Otter C.J., Tchicaya T., Schutte A.M. (1991). Effects of age, sex and hunger on the antennal olfactory sensitivity of tsetse flies. Physiol. Entomol..

[42] Germinara G.S., Ganassi S., Pistillo M.O., Di Domenico C., De Cristofaro A., Di Palma A.M. (2017). Antennal olfactory responses of adult meadow spittlebug, *Philaenus spumarius*, to volatile organic compounds (VOCs). PLoS ONE.

[43] Germinara G.S., De Cristofaro A., Rotundo G. (2009). Antennal olfactory responses to individual cereal volatiles in *Theocolax elegans* (Westwood) (Hymenoptera: Pteromalidae). J. Stored Prod. Res..

[44] Yang H., Yang W., Liang X.Y., Yang M.F., Yang C.P., Zhu T.H., Wu X.L. (2011). The EAG and behavioral responses of *Batocera horsfieldi* (Coleoptera: Cerambycidae) to the composition of volatiles. J. Kans. Entomol. Soc..

[45] Elgar A., Zhang D., Wang Q., Wittwer B., Pham H.T., Johnson T.L., Freelance C.B., Coquilleau M. (2018). Insect antennal morphology: The evolution of diverse solutions to odorant perception. Yale J. Biol. Med..

[46] Barata E.N., Pickett J.A., Wadhams L.J., Woodcock C.M., Mustaparta H. (2000). Identification of host and nonhost semiochemicals of eucalyptus woodborer *Phoracantha semipunctata* by gas chromatography-electroantennography. J. Chem. Ecol..

[47] Lopes O., Barata N.E., Mustaparta H., Araújo J. (2002). Fine structures of antennal sensilla basiconica and their detection of plant volatiles in the eucalyptus woodborer, *Phoracantha semipunctata* Fabricius (Coleoptera, Cerambycidae). Arthropod Struct. Dev..

[48] Silk P.J., Sweeney J., Wu J., Price J., Gutowski J.M., Kettela E.G. (2007). Evidence for a male-produced pheromone in *Tetropium fuscum* (F.) and *Tetropium cinnamopterum* (Kirby) (Coleoptera: Cerambycidae). Naturwissenschaften.

[49] Yang H., Yang W., Yang M.F., Yang C.P., Zhu T.H., Huang Q. (2010). Effects of plant volatiles on the EAG and behavioral responses of *Batocera horsfieldi* Hope (Coleoptera: Cerambycidae). J. Agric. Urban Entomol..

[50] Raguso R.A., Light D.M., Pickersky E. (1996). Electroantennogram responses of *Hyles lineata* (Onagraceae) and other moth-pollinated flowers. J. Chem. Ecol..

[51] El-Sayed A.M. (2019). The Pherobase: Database of Insect Pheromones and Semiochemicals. http://www.pherobase.com.

[52] Prokopy R.J., Phelan P.L., Wright S.E., Minalga A.J., Barger R., Leskey T.C. (2001). Compounds from host fruit odor attractive to adult plum curculios (Coleoptera: Curculionidae). J. Entomol. Sci..

[53] Schorkopf D.L.P. (2016). Male meliponine bees (*Scaptotrigona* aff. *depilis*) produce alarm pheromones to which workers respond with fight and males with flight. J. Comp. Physiol. A.

[54] Torto B., Suazo A., Alborn H., Tumlinson J.H., Teal P.E.A. (2005). Response of the small hive beetle (*Aethina tumida*) to a blend of chemicals identified from honeybee (*Apis mellifera*) volatiles. Apidologie.

[55] Mikolajczak K.L., Zilkowski B.W., Smith C.R., Burkholder W.E. (1984). Volatile food attractants for *Oryzaephilus surinamensis* (L.) from oats. J. Chem. Ecol..

[56] McGraw B.A., Rodriguez-Saona C., Holdcraft R., Szendrei Z., Koppenhfer A.M. (2011). Behavioral and electrophysiological responses of *Listronotus maculicollis* (Coleoptera: Curculionidae) to volatiles from intact and mechanically damaged annual bluegrass. Environ. Entomol..

[57] Dettner K., Reissenweber F. (1991). The defensive secretion of Omaliinae and Proteininae (Coleoptera: Staphylinidae): Its chemistry, biological and taxonomic significance. Biochem. Syst. Ecol..

[58] Huber D.P.W., Gries R., Borden J.H., Pierce H.D. (2000). A survey of antennal responses by five species of coniferophagous bark beetles (Coleoptera: Scolytidae) to bark volatiles of six species of angiosperm trees. Chemoecology.

[59] Obeng-Ofori D., Torto B., Njagi P.G.N., Hassanali A., Amiani H. (1994). Fecal volatiles as part of the aggregation pheromone complex of the desert locust, *Schistocerca gregaria* (Forskal) (Orthoptera: Acrididae). J. Chem. Ecol..

[60] Molnár B.P., Tóth Z., Kárpáti Z. (2017). Synthetic blend of larval frass volatiles repel oviposition in the invasive box tree moth, *Cydalima perspectalis*. J. Pest Sci..

[61] Byrne K.J., Swigar A.A., Silverstein R.M., Borden J.H., Stokkink E. (1974). Sulcatol: Population aggregation pheromone in the scolytid beetle, *Gnathotrichus sulcatus*. J. Insect Physiol..

[62] Borden J.H., McLean J.A. (1979). Secondary attraction in *Gnathotricus retusus* and cross attraction of *G. sulcatus* (Coleoptera: Scolytidae). J. Chem. Ecol..

[63] Fletchmann C.A.H., Berisford C.W. (2003). Identification of sulcatol, a potential pheromone of the ambrosia beetle *Gnathotricus materiarus* (Col., Scolytidae). J. Appl. Entomol..

[64] Quiroz A., Petterson J., Pickett J.A., Wadhams L.J., Niemeyer H.M. (1997). Semiochemicals mediating spacing behavior of bird cherry-oat aphid, *Rhopalosiphum padi* feeding on cereals. J. Chem. Ecol..

[65] Gatti Liguori P., Zerba E., Gonzalez Audino P. (2011). Anatomical site of pheromone accumulation and temporal pattern of pheromone emission in the ambrosia beetle, *Megaplatypus mutatus*. Physiol. Entomol..

[66] Algarvio R., Teixeira C., Barata E., Pickett J., Novas P.C., Figueiredo D. Identification of a putative aggregation pheromone from males Platypus cylindrus (Coleoptera: Platypodidae). Proceedings of the 19th Annual Meeting International Society of Chemical Ecology.

[67] Borden J.H., Wilson I.M., Gries R., Chong L.J., Pierce H.D., Gries G. (1998). Volatiles from the bark of trembling aspen, *Populus tremuloides* Michx. (Salicaceae) disrupt secondary attraction by the mountain pine beetle, *Dendroctonus ponderosae* Hopkins (Coleoptera: Scolytidae). Chemoecology.

[68] Bernays E.A., Oppenheim S., Chapman R.F., Kwon H., Gould F. (2000). Taste sensitivity of insect herbivores to deterrents is greater in specialists than in generalists: A behavioural test of the hypothesis with two closely related caterpillars. J. Chem. Ecol..

[69] Linn C., Nojima S., Roelofs W. (2005). Antagonistic effects of nonhost fruit volatiles on chemically-mediated discrimination of host fruit by *Rhagoletis pomonella* flies infesting apple, hawthorn (*Crataegus* spp.) and flowering dogwood (*Cornus florida*). Èntomol. Exp. Appl..

[70] Germinara G.S., De Cristofaro A., Rotundo G. (2011). Chemical cues for host location by the chestnut gall wasp, *Dryocosmus kuriphilus*. J. Chem. Ecol..

